# Transcriptomic Profiles for Elucidating Response of Bladder Intracavitary Hyperthermic Perfusion Chemotherapy in High‐Risk Nonmuscular Invasive Bladder Cancer

**DOI:** 10.1002/cam4.70672

**Published:** 2025-02-20

**Authors:** Zhicheng Huang, Tianhui Zhang, Jinghua Pan, Guihao Zhang, Linjun Jiang, Huiming Jiang, Pei Wan, Ying Peng, Wenchao Zou, Qinghua Liu, Nanhui Chen

**Affiliations:** ^1^ Shantou University Medical College Meizhou Clinical College Meizhou Guangdong Province China; ^2^ Department of Urology Meizhou People's Hospital Meizhou Guangdong Province China; ^3^ Department of Magnetic Resonance Imaging Meizhou People's Hospital Meizhou Guangdong Province China; ^4^ Department of General Surgery The First Affiliated Hospital of Jinan University Guangzhou China; ^5^ Department of Pathology Meizhou People's Hospital Meizhou Guangdong Province China

**Keywords:** bladder cancer, immune response, intracavitary hyperthermic perfusion chemotherapy, resistance, transcriptomics

## Abstract

**Background:**

Bladder intracavitary hyperthermic perfusion chemotherapy (HIPEC) is a promising treatment for non‐muscular invasive bladder cancer (NMIBC). However, the molecular mechanisms underlying the response to HIPEC remain poorly understood. This study aimed to elucidate the transcriptomic profiles associated with the response to HIPEC in NMIBC patients.

**Methods:**

RNA sequencing was performed on bladder tumor samples from NMIBC patients who underwent HIPEC treatment. Differentially expressed genes (DEGs) between responders and non‐responders to HIPEC were identified. Gene ontology and pathway analysis were conducted to explore the biological functions and pathways enriched in the DEGs. Additionally, the expression of specific immune‐related genes was evaluated for their association with HIPEC response. The diagnostic efficiency of selected genes in predicting relapse before and after HIPEC treatment was assessed in a validation cohort.

**Results:**

We assessed the expression status of differentially expressed genes (DEGs) between responders and non‐responders to HIPEC. Gene ontology and pathway analysis revealed that DEGs were enriched in immune‐related pathways, including cytokine‐cytokine receptor interaction, chemokine signaling pathway, and antigen processing and presentation. Furthermore, the expression of several immune‐related genes, including ZMAP4, UPP2, and GALR1, was significantly associated with the response to HIPEC. Therefore, the immune system's reaction plays a crucial role in the response to HIPEC in patients with NMIBC. At last, a considerable diagnostic efficiency that tissue TMEFF2, KRT222, and GTSF1 in predicting relapse in NMIBC patients after HIPEC treatment, and ZMAP4, UPP2, and GALR1 in predicting relapse in NMIBC patients before HIPEC treatment in the validation cohort.

**Conclusion:**

Transcriptomic profiling revealed that immune‐related pathways and genes play a crucial role in the response to HIPEC in NMIBC patients. These findings suggest that transcriptomic profiling could provide a valuable tool for predicting treatment outcomes and identifying therapeutic targets for NMIBC.

## 1 Introduction

Bladder cancer is the ninth most common cancer worldwide, with approximately 550,000 patients newly diagnosed annually. NMIBC accounts for approximately 75% of all bladder cancer cases. It is characterized by the presence of cancer cells in the bladder lining without invasion into the muscle layer [1]. Although transurethral resection of the bladder tumor (TURBt) is the standard treatment for NMIBC, the recurrence rate in high‐risk NMIBC patients is frequently high. Up to 70% of high‐risk NMIBC patients experience tumor recurrence within 5 years after TURBt surgery [2].

Bladder intracavitary HIPEC is a promising treatment option for NMIBC [3]. HIPEC involves the direct administration of chemotherapy drugs into the bladder, followed by the application of heat to boost drug penetration and amplify the cytotoxic effects of the drugs [4]. Several clinical studies [5, 6] have reported promising outcomes with HIPEC, including high response rates and low recurrence rates. However, the molecular mechanisms responsible for the efficacy of HIPEC in high‐risk NMIBC remain unclear.

Understanding the molecular mechanisms of HIPEC in high‐risk NMIBC is crucial for optimizing treatment strategies and improving patient outcomes. Innovative diagnostic approaches, such as liquid biopsies, utilize molecular biomarkers found in urine or blood samples, offering a noninvasive method for monitoring disease progression and treatment response [7, 8, 9]. Moreover, the integration of next‐generation sequencing allows for the identification of specific mutations that can guide targeted therapies, improving patient outcomes [10, 11]. In this study, we performed high‐throughput RNA sequencing (RNA‐Seq) to determine the molecular mechanisms of HIPEC in NMIBC, including the molecular change before and after the treatment of HIPEC in high‐risk NMIBC, and the molecular characteristics between the recurrent and non‐recurrent NMIBC patients after HIPEC treatment. We also analyzed the changes in immune infiltration and immune microenvironment in NMIBC after HIPEC treatment. Overall, this study aimed to provide a comprehensive overview of the molecular mechanisms of HIPEC in high‐risk NMIBC and highlight the areas for future research.

## 2 Materials and Methods

### 2.1 Ethics Statement

This clinical research scheme was authorized by the Meizhou People's Hospital Clinical Scientific Research and New Technology Ethics Committee (Huangtang Hospital, Meizhoulunshen 2021‐C‐29, July 22, 2021). It was performed in accordance with the Declaration of Helsinki (1975) and the International Ethical Guidelines for Biomedical Research Involving Human Subjects (2002). Before specimen collection, written informed consent was obtained from all participants.

### 2.2 Patients, Clinical Samples, and Data

Overall, 20 patients histopathologically diagnosed with NMIBC at the Meizhou Peoplés Hospital (Huangtang Hospital) from March 2017 to May 2021 were included in this study for RNA‐sequencing. Additionally, 43 patients from June 2021 to February 2024 were included for the external validation cohort. Patients (1) who were diagnosed with NMIBC by histopathological examination; (2) who were initially diagnosed with and treated for NMIBC, but with no history of radiotherapy and chemotherapy; (3) with complete clinicopathological and follow‐up data; and (4) who signed an informed consent form were included in the study. In contrast, patients (1) who developed complications with other malignant tumors; (2) whose NMIBC was complicated with acute cystitis, urethral stricture, and abnormal coagulation function; and (3) developed severe liver and kidney dysfunction.

**FIGURE 1:**
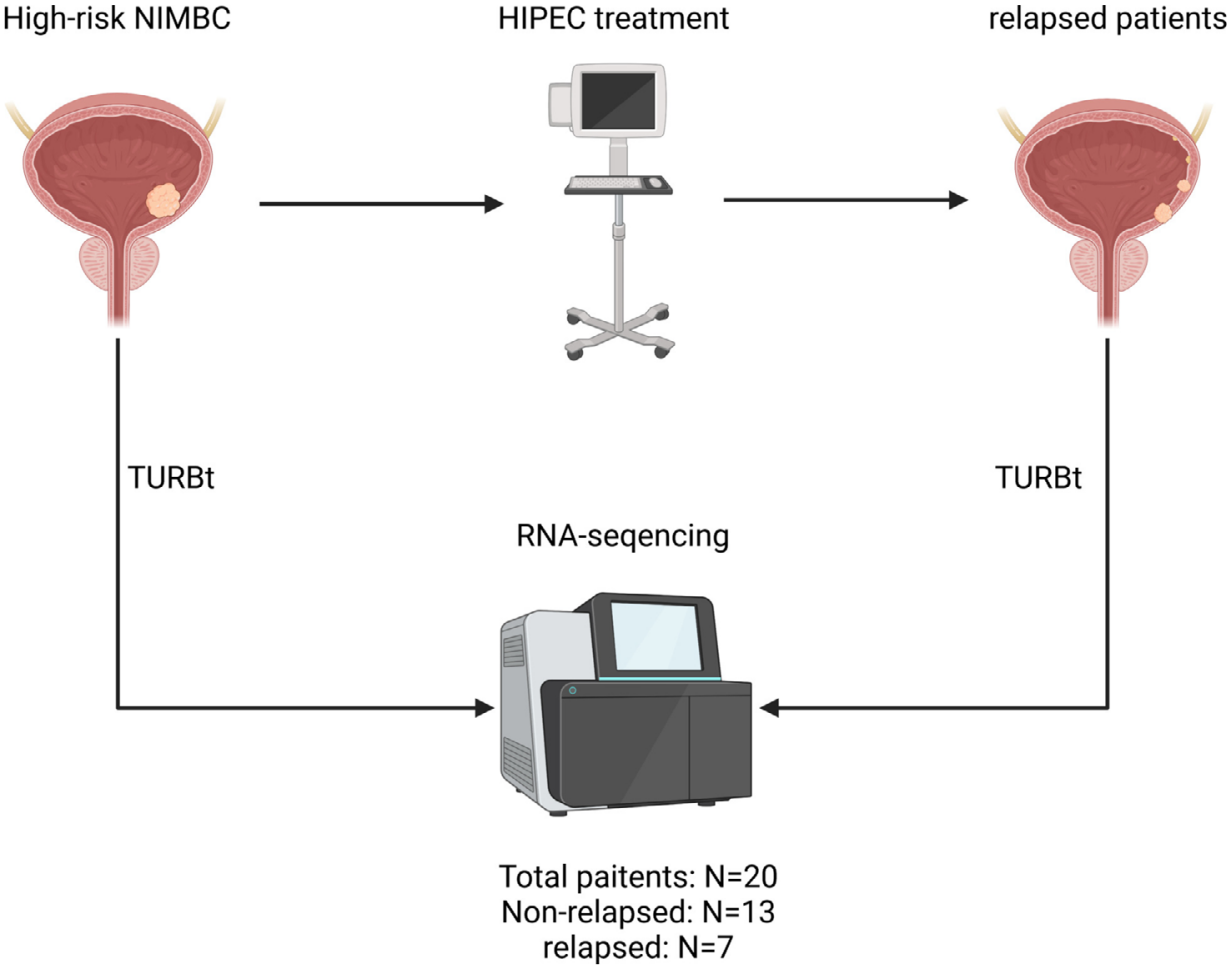
Flowchart of the TURBt, HIPEC, and RNA sequencing in high‐risk NMIBC patients. All included NMIBC patients (*n* = 20) underwent TURBt treatment (samples were obtained before any treatment (*n* = 20): 13 for the non‐relapsed group and 7 for the relapsed group (Pre‐HIPEC group)). Then, all patients received HIPEC treatment to reduce relapse, and seven patients with recurrence subsequently underwent TURBt treatment to obtain samples (*n* = 7, Post‐HIPEC group).

Data on age, gender, tumor node metastasis (TNM) stage, histologic grade, tumor number, tumor size, and degree of differentiation of all included patients were recorded. A total of 20 NMIBC patients were analyzed; of them, seven patients experienced relapse, while 13 patients did not experience relapse after HIPEC treatment. Table 1 presents the baseline clinical features of the relapsed group and non‐relapsed group. After TURBt treatment, tumor tissues were collected from the patients with NMIBC, respectively, during surgery and immediately placed in liquid nitrogen. The clinical specimens were then stored in a −80 °C refrigerator for subsequent RNA‐Seq. One sample will be divided into two parts: one for RNA‐Seq and the other for rt‐qPCR. The specific processing flow is shown in Figure 1.

**TABLE 1:** Comparative analysis of clinical baseline data.

Clinicopathological variables	Relapsed group (*n* = 7)	Non‐relapsed group (*n* = 13)	*p*
Gender			0.587
Male	5	11	
Female	2	2	
Preoperative age (years)			0.651
≤ 60	4	9	
> 60	3	4	
Tumor size (cm)			> 0.999
≤ 3	5	9	
> 3	2	4	
Amount of tumors			0.656
≤ 3	6	8	
> 3	2	5	
Grade			0.651
Low	4	9	
High	3	4	
T stage			0.587
Ta	5	11	
T1	2	2	

### 2.3 TURBt and HIPEC Treatment

After the initial diagnosis, all patients underwent TURBt following the standard protocols. Within 1 week after the surgical procedure, appropriate chemotherapeutic agents were selected for intravesical hyperthermic chemotherapy. High‐risk NMIBC patients who have undergone TURBt received hyperthermic intracavitary perfusion treatment using the BR‐TRG‐II system. The perfusion system is configured to operate in a circulation mode, maintaining the inflow temperature at 45.0°C ± 0.3°C and the outflow temperature at 43.0°C ± 0.5°C, while regulating the perfusion rate between 100 and 200 mL/min. The perfusion rate may be adjusted based on individual patient tolerance, maintaining an appropriate perfusion pressure to ensure optimal bladder filling. The duration of hyperthermic perfusion treatment approximates 45 min. Intravesical hyperthermic chemotherapy with PiRubicin was administered at a dose of 15–30 mg/m^2^, adjusted according to body surface area, and diluted to a concentration of 500 μg/mL. Treatment was continuously administered for 12 months, once a week during the initial 2 months and once a month for another 10 months. In patients with tumor recurrence, a repeat TURBt was performed, and the excised tissue was utilized for RNA‐seq of the recurrent NMIBC tissue.

### 2.4 Total RNA Isolation, Library Preparation, and Sequencing

Fourteen samples from the relapsed group (seven for pre‐HIPEC and seven for post‐HIPEC) and 13 samples from the non‐relapsed group were submitted for RNA‐Seq. The total RNA was extracted using the Qiagen RNeasy formalin‐fixed paraffin‐embedded kit (73,504, Qiagen, Hilden, Germany) in accordance with the manufacturer's protocol. The purity and quantity of the obtained total RNA were determined using the NanoDrop spectrophotometer. The integrity of the RNA was analyzed using the RNA Nano6000 Assay Kit on the Bioanalyzer 2100 system (Agilent Technologies, CA, USA). Approximately 1 μg of RNA per sample was utilized as input for the subsequent RNA sample preparations. Strand‐specific RNA‐Seq libraries were then generated utilizing the Whole RNA‐seq Lib Prep kit for Illumina (RK20303, ABclonal, Shanghai, China). The quality of the resulting libraries was evaluated using the Agilent Bioanalyzer 2100 system (Agilent, USA). Ultimately, the prepared libraries were subjected to sequencing at the Novogene Bioinformatics Institute (Beijing, China) on an Illumina HiseqX10 platform using a 150‐bp paired‐end read protocol.

### 2.5 Gene Expression Quantification

The initial RNA‐Seq reads underwent filtering using FastQC. Subsequently, the reads were aligned to the Ensembl human genome assembly using STAR (v2.7.0 f) with default parameters. The gene expression levels were measured using both raw count and transcripts per kilobase million. The annotation information of the mRNA within the human genome was retrieved from the GENCODE (v19) database.

### 2.6 Analysis of Differentially Expressed Genes (DEGs) and Enrichment of Signaling Pathways

Three groups of patients underwent differential expression analysis: (1) seven with relapse NMIBC (pre‐HIPEC) and 13 with non‐relapse NMIBC and (2) seven with pre‐HIPEC and seven post‐HIPEC. The R package “DESeq2” was used for determining the expression status of DEGs based on a cutoff *p* ≤ 0.05 and |a log2FoldChange| of ≥ 1. The upregulated genes and downregulated genes were imported into the “clusterProfiler” software for ontology and pathway enrichment analysis utilizing the Gene Ontology and KEGG databases.

### 2.7 Calculation of the Immune and Stromal Scores and Estimation

The Estimation of STromal and Immune cells in MAlignant Tumor tissues using Expression (ESTIMATE) algorithm is a computational approach used to estimate the fraction of tumor purity in malignant tissues by leveraging the expression data of immune cells and stromal cells. In this study, we employed the ESTIMATE algorithm to assess the immune grade, stromal grade, and tumor purity of patients with NMIBC, considering the Immunity_H, Immunity_M, and Immunity_L groups as reference points. To further investigate the composition of immune cell types within each subset, we utilized the CIBERSORT (available at https://cibersortx.stanford.edu/), EPIC, and quanTIseq method, a biological approach for cell‐type identification by estimating relative subsets of RNA transcripts. The CIBERSORT package was employed to calculate the distribution of immune cell types within each subset. A comparison of immune cell proportions among NMIBC subtypes was performed using the Kruskal–Wallis test. The ESTIMATE and CIBERSORT packages in R (version 3.6.2, available at https://www.R‐project.org/) were utilized for the analyses.

### 2.8 qRT‐PCR

The total RNA was extracted from cells with TRIzol reagent (Invitrogen, Carlsbad, CA, USA). cDNA was generated according to the manufacturer's protocol (Takara). Quantitative reverse transcription polymerase chain reaction (qRT‐PCR) was performed using an Applied Biosystems instrument at a reaction volume of 20 μL (SYBR Green Real‐Time PCR Master Mix; Tsingke, Beijing, China). The gene GAPDH was used as an internal parameter [12]. All samples were tested in triplicate. qRT‐PCR primers for this study are listed in Table S1.

### 2.9 Statistics

All analyses were conducted using the R environment (v3.6.0). All comparisons for continuous variables were performed using the two‐sided Mann–Whitney U test for two groups, and the differential expression gene (DEG) comparisons between pre‐ and post‐HIPEC treatment groups used a paired test design. TMEFF2, KRT222, and GTSF1 were evaluated for predicting relapse in NMIBC patients after HIPEC treatment, while ZMAP4, UPP2, and GALR1 were assessed for predicting relapse in NMIBC patients before HIPEC treatment through ROC curve analysis. A *p* < 0.05 was considered significant. The Benjamini–Hochberg method was used for multiple testing correction.

## 3 Results

### 3.1 RNA‐Sequencing Reveals DEGs of in Relapsed NMIBC Patients After HIPEC Treatment

**FIGURE 2:**
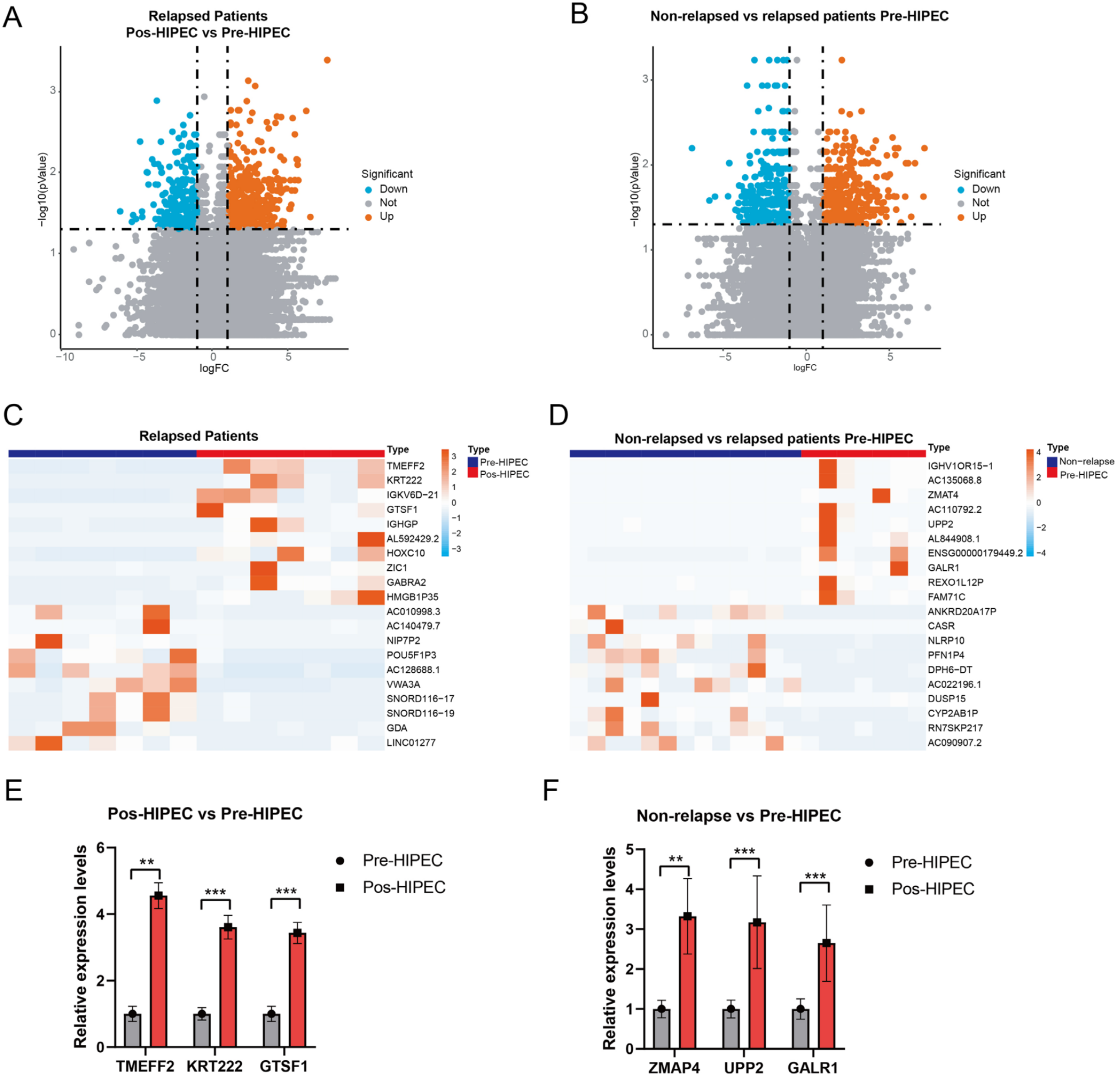
(A, B) The volcano plot showed the differentially expressed genes (DEGs) detected in the pre‐HIPEC and post‐HIPEC relapsed and non‐relapsed NMIBC groups. (C, D) Heatmaps illustrated the DEGs detected between the pre‐HIPEC and post‐HIPEC relapsed NMIBC groups, as well as the relapsed and non‐relapsed NMIBC samples. (E) qRT‐PCR was performed to further verify the markers in the pre‐HIPEC and post‐HIPEC relapsed NMIBC groups, including TMEFF2, KRT222, and GTSF1. (F) qRT‐PCR was performed to further verify the markers in the relapsed and non‐relapsed NMIBC samples, including ZMAT4, UPP2, and GALR1. **p < 0.01, ***p < 0.001.

There is no significant difference in clinical baseline characteristics between the relapsed and non‐relapsed groups (Table 1). To determine the underlying mechanism of NMIBC relapse in patients who underwent HIPEC treatment, we compared the expression status of DEGs in the high‐risk NMIBC pre‐ and post‐HIPEC treatment groups. A total of 1277 DEGs (*p* < 0.05) were identified in NMIBC patients who experienced relapse before and after HIPEC treatment. The top 20 differentially expressed markers are shown in Table 2. Furthermore, volcano plots and heatmaps were generated to visually represent the DEGs detected in the pre‐HIPEC and post‐HIPEC groups (Figure 2A,C). After HIPEC treatment, the NMIBC tissue had higher mRNA expression levels of TMEFF2, KRT222, IGKV6D−21, GTSF1, IGHGP, AL592429.2, HOXC10, ZIC1, GABRA2, and HMGB1P35.

**TABLE 2:** Top 20 different expression markers between pre‐HIPEC and post‐HIPEC treatment in relapsed group.

Gene	pre‐HIPEC	post‐HIPEC	logFC	*p*
SCAMP2	26.27857143	17.92	−0.552316212	0.000582751
TMEM63A	87.41428571	39.26857143	−1.154493958	0.000582751
SNORA3A	635.8757143	245.7871429	−1.371335359	0.000582751
AC019097.1	0.312857143	1.387142857	2.148540426	0.000582751
AC092620.1	3.052857143	0.652857143	−2.225320838	0.000582751
AP006284.1	2.874285714	0.332857143	−3.110228445	0.000582751
AL049828.1	3.071428571	0.924285714	−1.732499042	0.000582751
CACNA1I	1.534285714	0.457142857	−1.746850183	0.001165501
C3orf52	23.22714286	6.341428571	−1.872931914	0.001165501
MUC1	47.83285714	4.077142857	−3.552371556	0.001165501
ENSG00000197681.8	10.40571429	1.66	−2.648120854	0.001165501
ZNF321P	16.39857143	6.79	−1.272086659	0.001165501
AC006077.2	6.35	1.267142857	−2.325177409	0.001165501
DNAH5	1.065714286	0.224285714	−2.248411071	0.00214071
CDK18	12.55142857	5.892857143	−1.09081238	0.002331002
TICAM1	4.241428571	2.64	−0.684012337	0.002331002
SGPP2	10.64714286	1.44	−2.886325621	0.002331002
ZNF846	19.64	7.354285714	−1.417137798	0.002331002
IGHG1	52.00714286	522.79	3.329449858	0.002331002
RNPS1P1	2.305714286	0.872857143	−1.401396293	0.002331002

Moreover, to identify the genetic characteristics in relapsed NMIBC patients after HIPEC treatment, a comparison of the DEGs was conducted between the pre‐HIPEC and non‐relapsed NMIBC groups. A total of 1158 DEGs (*p* < 0.05) were identified in the relapsed and non‐relapsed NMIBC patients before HIPEC treatment. The top 20 differentially expressed markers are shown in Table 3. Relapsed NMIBC patients (pre‐HIPEC) exhibited higher expression levels of IGHV1OR15–1, AC135068.8, ZMAT4, AC110792.2, UPP2, AL844908.1, ENSG00000179449.2, GALR1, REXO1L12P, and FAM71C compared with non‐relapsed NMIBC patients (Figure 2B,D). These findings depict the transcriptomic profiles of relapsed NMIBC patients before and after HIPEC treatment.

**TABLE 3:** Top 20 different expression markers between relapsed and non‐relapsed groups before HIPEC.

Gene	Non‐relapsed group	Relapsed group	LogFC	*p*
IGHV1OR15–1	0.01	1.984285714	7.632475962	0.000410399
AC135068.8	0.01	1.984285714	7.632475962	0.000410399
COL23A1	0.049230769	0.257142857	2.384937892	0.000736858
AC110760.2	0.142307692	1.022857143	2.845519113	0.000856032
RN7SK	84322.65462	58139.38429	−0.536404493	0.001160991
AL138878.2	0.275384615	0.021428571	−3.683840386	0.001299949
RPL3P6	0.116153846	0.567142857	2.287675254	0.001316934
ADGRE2	1.157692308	4.004285714	1.790293053	0.001702786
RP9P	0.829230769	1.941428571	1.227273074	0.001702786
AC142381.3	2.618461538	8.525714286	1.703101295	0.001702786
AC110792.2	0.012307692	0.925714286	6.232934799	0.001736615
AC009299.2	0.119230769	0.748571429	2.650383392	0.001828466
MFSD1P1	0.478461538	0.171428571	−1.480795378	0.001967264
ENSG00000229198.2	0.192307692	3.771428571	4.293622726	0.002030676
CLEC17A	0.027692308	0.655714286	4.565510138	0.002065182
AC114760.2	0.035384615	1.425714286	5.332418845	0.002130761
FSCN1P1	0.039230769	0.221428571	2.496783859	0.002303125
DIO3	0.024615385	0.302857143	3.621005251	0.00238704
PDE9A	3.554615385	8.212857143	1.208190737	0.002425181
HNRNPA1P62	0.005384615	0.098571429	4.194254331	0.002442745

Finally, we employed qRT‐PCR to validate the expression of selected markers. We observed a significant increase in the expression levels of TMEFF2, KRT222, and GTSF1 after HIPEC treatment in the relapse cohort, suggesting that HIPEC treatment mediates the upregulation of these genes (Figure 2E). Additionally, ZMAP4, UPP2, and GALR1 showed significantly higher expression in the pre‐HIPEC patients in the relapse cohort compared with the non‐relapsed patients (Figure 2F). These findings indicate potential markers for predicting the response of HIPEC treatment in NMIBC patients. Moreover, these results affirm the consistency of the RNA‐seq and qRT‐PCR findings.

### 3.2 GO and KEGG Analysis of DEGs in Relapsed NMIBC Patients

**FIGURE 3:**
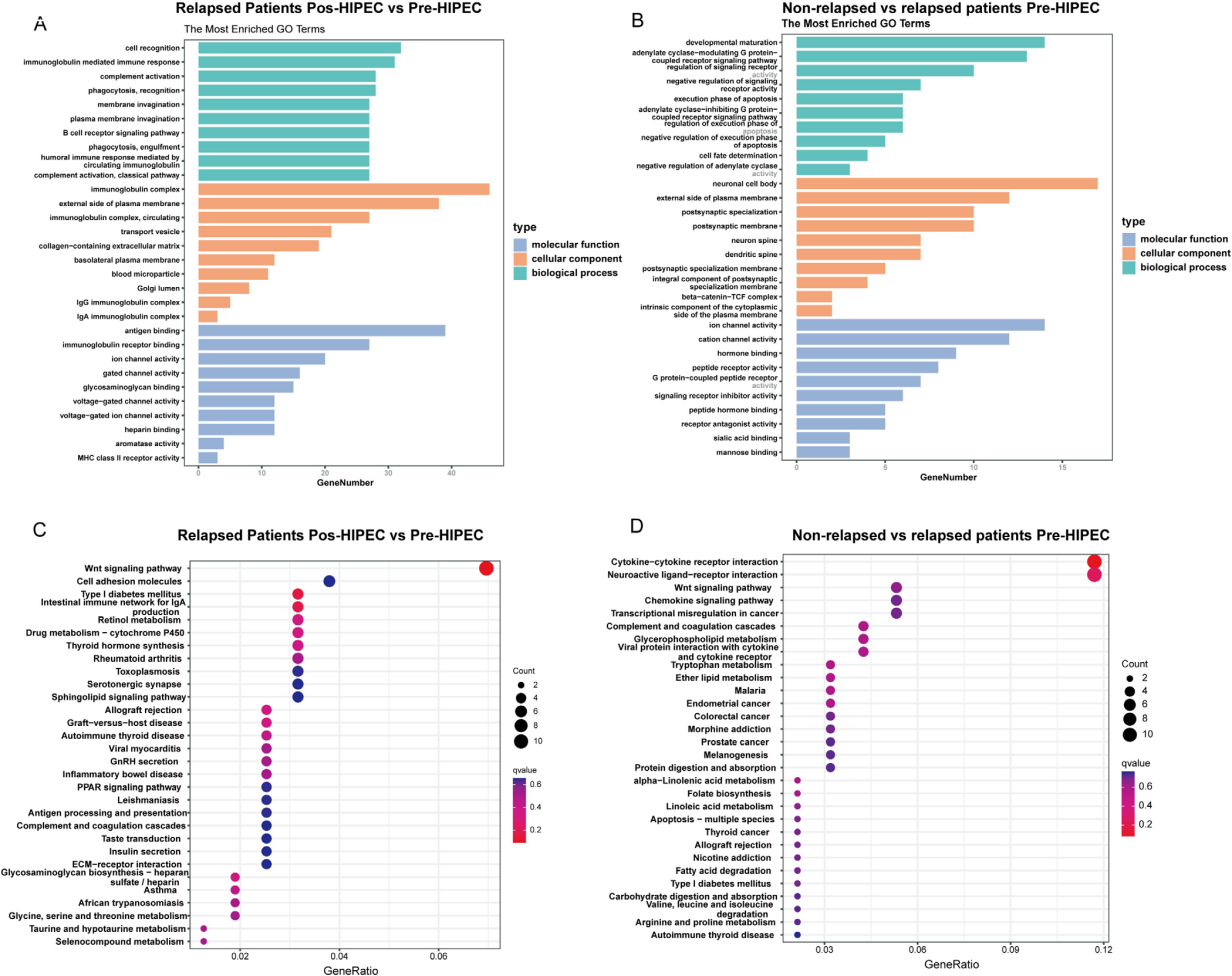
(A) Gene Ontology (GO) enrichment analysis of differentially expressed genes (DEGs) between pre‐HIPEC and post‐HIPEC in relapsed NMIBC samples. (B) GO enrichment analysis of DEGs between relapsed and non‐relapsed NMIBC samples. (C) Kyoto Encyclopedia of Genes and Genomes (KEGG) enrichment analysis of DEGs between pre‐HIPEC and post‐HIPEC in relapsed NMIBC samples. (D) KEGG enrichment analysis of DEGs between relapsed and non‐relapsed NMIBC samples.

To elucidate the functional implications of differentially expressed genes (DEGs) in relapsed NMIBC samples, Gene Ontology (GO) and Kyoto Encyclopedia of Genes and Genomes (KEGG) analyses were conducted on the identified DEGs. In the context of relapsed NMIBC samples, the DEGs identified before and after HIPEC treatment were predominantly enriched in various biological processes, including cell recognition, immunoglobulin‐mediated immune response, complement activation, phagocytosis, and others. The enrichment analysis of cellular components and molecular functions is depicted in Figure 3A. In relapsed and non‐relapsed NMIBC samples, the DEGs were primarily enriched in developmental maturation, the activation of the adenylate cyclase‐modulating G protein‐coupled receptor signaling pathway, and the regulation of signaling receptor activity. The cellular component and molecular function enrichments are illustrated in Figure 3B.

In the KEGG enrichment analysis, the DEGs identified in relapsed NMIBC samples before and after HIPEC treatment were associated with various pathways, including the Wnt signaling pathway, cell adhesion molecules, type I diabetes mellitus, and the intestinal immune network for IgA production (Figure 3C). Moreover, the DEGs distinguishing relapsed from non‐relapsed NMIBC samples were implicated in cytokine–cytokine receptor interaction, neuroactive ligand–receptor interaction, and the Wnt signaling pathway (Figure 3D). Collectively, these findings provide robust evidence supporting the pivotal role of the Wnt signaling pathway as a crucial molecular characteristic in relapsed NMIBC samples.

### 3.3 Immunoscore Analysis in Relapsed and Non‐Relapsed NMIBC Samples

**FIGURE 4:**
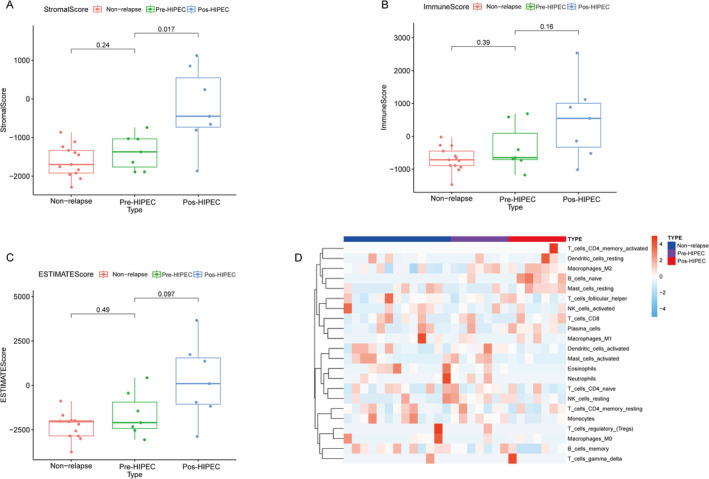
Immunoscore analysis of differentially expressed genes (DEGs) in relapsed and non‐relapsed NMIBC samples. (A) ESTIMATE analysis of stromal scores in different groups; (B) ESTIMATE analysis of immune scores in different groups; (C) ESTIMATE analysis of overall ESTIMATE scores in different groups; (D) CIBERSORT analysis of immune cell scores in different groups.

To further examine the changes in the tumor microenvironment (TME) within the samples of relapsed and non‐relapsed NMIBC patients, we conducted an ESTIMATE analysis of different samples. The NMIBC samples after HIPEC treatment showed a higher Stromal Score compared with the non‐relapsed NMIBC samples and the relapsed NMIBC samples before HIPEC treatment. However, no significant differences were observed in the total immune scores and ESTIMATE score between non‐relapsed, pre‐HIPEC, and post‐HIPEC relapsed NMIBC samples. Results of the CIBERSORT analysis of immune cell scores in different groups are shown in Figure 4D.

### 3.4 Comparison of Immune Infiltration Levels in Relapsed and Non‐Relapsed NMIBC Samples

**FIGURE 5:**
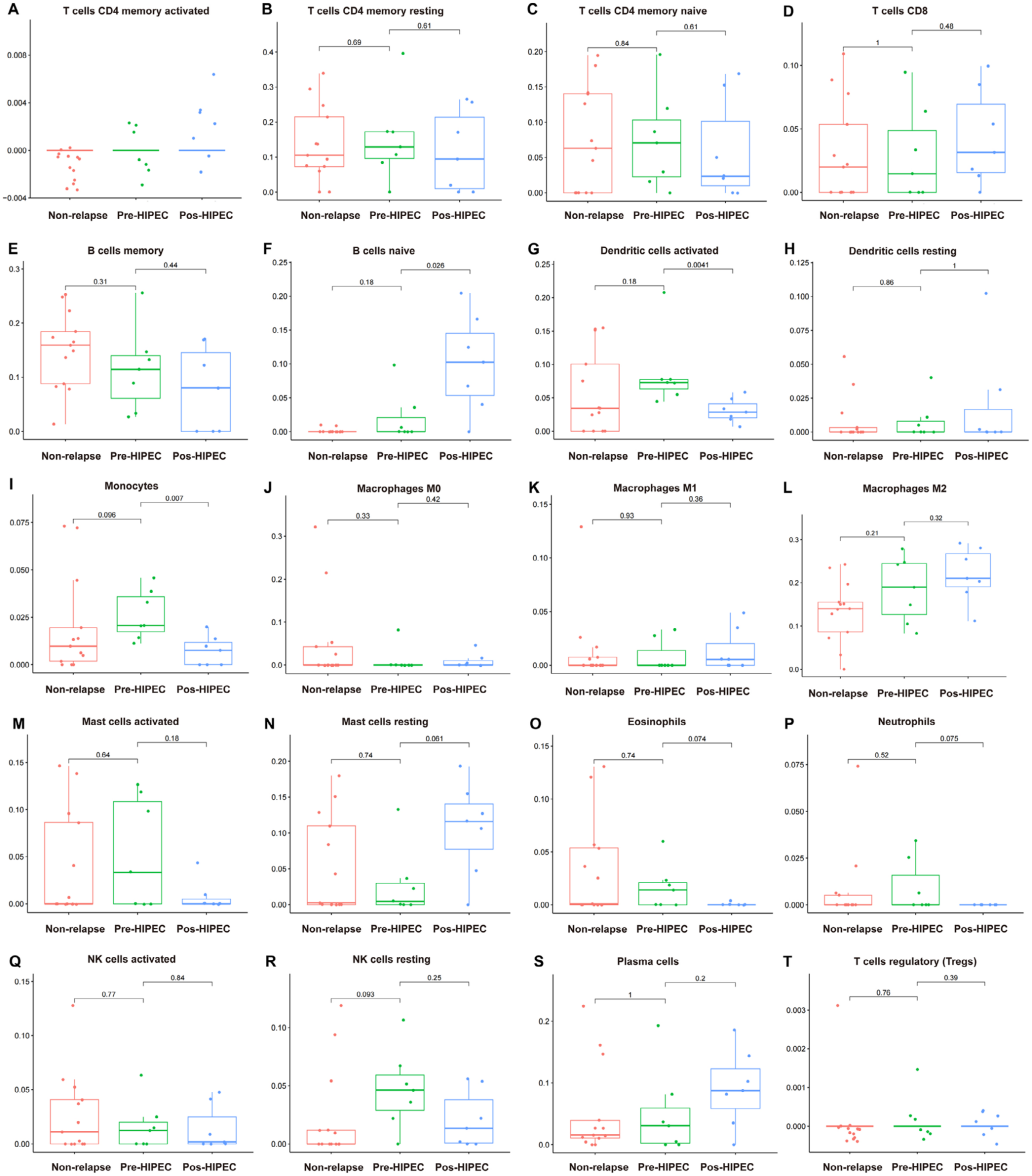
Differences in immune cell infiltration scores among different groups via CIBERSORT analysis. (A) Activated memory CD4 T cells; (B) resting memory CD4 T cells; (C) native CD4 T cells; (D) CD8 T cells; (E) memory B cells; (F) native B cells; (G) activated dendritic cells; (H) resting dendritic cells; (I) monocytes; (J) M0 macrophages; (K) M1 macrophages; (L) M2 macrophages; (M) activated mast cells; (N) resting mast cells; (O) eosinophils; (P) neutrophils; (Q) activated NK cells; (R) resting NK cells; (S) plasma cells; and (T) regulatory T cells.

To elucidate the alterations in the tumor microenvironment (TME) within relapsed NMIBC samples, we investigated immune infiltration levels in both relapsed and non‐relapsed NMIBC samples using CIBERSORT analysis. No significant differences were observed in the infiltration levels of activated memory CD4 T cells; resting memory CD4 T cells; native CD4 T cells; CD8 T cells; memory B cells; resting dendritic cells; M0 macrophages; M1 macrophages; M2 macrophages; activated mast cells; resting mast cells; eosinophils; neutrophils; activated natural killer (NK) cells; resting NK cells; plasma cells; and regulatory T cells (Tregs) (Figure 5A–E,H,J–T).

However, notable differences were observed in immune cell infiltration between relapsed NMIBC samples after HIPEC treatment. Specifically, these samples exhibited higher levels of native B‐cell infiltration (Figure 5F), along with lower levels of infiltrated activated dendritic cells (Figure 5G) and monocytes (Figure 5I). Moreover, we also perform the immune infiltration between the three groups in Figure S2 via EPIC and Figure S3 via quanTIseq method. We found the post‐HIPEC group has higher endothelial (Figure S2) and M2 macrophages (Figure S3). These findings provide further evidence of the dynamic changes occurring within the TME of relapsed NMIBC samples before and after HIPEC treatment.

### 3.5 Validation in the Validation Cohorts

**FIGURE 6:**
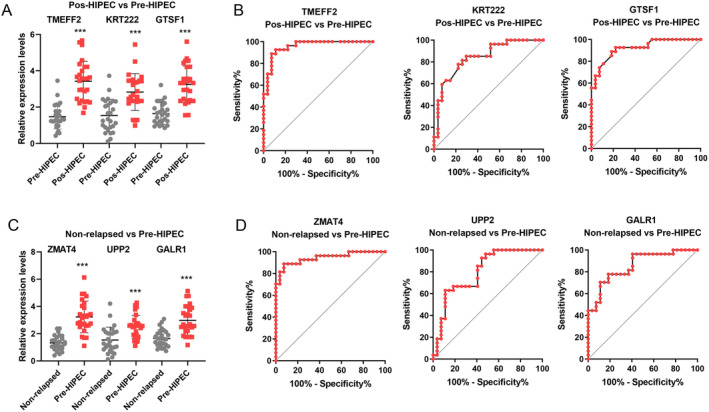
Validation of markers in the external validation cohort. (A) qRT‐PCR was performed to further verify the expression of markers in the pre‐HIPEC and post‐HIPEC relapsed NMIBC groups, including TMEFF2, KRT222, and GTSF1. (B) Diagnostic value of tissue TMEFF2, KRT222, and GTSF1 in predicting relapse in NMIBC patients after HIPEC treatment. (C) qRT‐PCR was performed to further verify the expression of markers in the relapsed and non‐relapsed NMIBC samples, including ZMAP4, UPP2, and GALR1. (D) Diagnostic value of tissue ZMAP4, UPP2, and GALR1 in predicting relapse in NMIBC patients before HIPEC treatment. ***p < 0.001.

Finally, we further collected a validation cohort that included 43 NMIBC patients. Of these, 16 patients experienced relapse, while 27 patients did not experience relapse after HIPEC treatment. Table 4 presents the baseline clinical features of the relapsed and non‐relapsed groups. We found a significant increase in the expression levels of TMEFF2, KRT222, and GTSF1 after HIPEC treatment in the relapse cohort (Figure 6A). The area under the curve (AUC) for predicting relapse in NMIBC patients after HIPEC treatment was as follows: TMEFF2: AUC = 0.956 (sensitivity 96.3%, specificity 77.78%, and cutoff point 1.875); KRT222: AUC = 0.842 (sensitivity 93.2%, specificity 48.15%, and cutoff point 1.245); GTSF1: AUC = 0.922 (sensitivity 94.7%, specificity 48.15%, and cutoff point 1.555) (Figure 6B).

**TABLE 4:** Comparative analysis of clinical baseline data in validation cohorts.

Clinicopathological variables	Relapsed group (*n* = 16)	Non‐relapsed group (*n* = 27)	*p*
Gender			0.7867
Male	13	21	
Female	3	6	
Preoperative age (years)			0.7799
≤ 60	7	13	
> 60	9	14	
Tumor size (cm)			0.6555
≤ 3	10	15	
> 3	6	12	
Amount of tumors			0.4155
≤ 3	12	17	
> 3	4	10	
Grade			0.6471
Low	8	12	
High	8	16	
T stage			0.7867
Ta	13	21	
T1	3	6	

Additionally, ZMAP4, UPP2, and GALR1 showed significantly higher expression in pre‐HIPEC patients in the relapse cohort compared with the non‐relapsed patients (Figure 6C). The AUC for predicting relapse in NMIBC patients before HIPEC treatment was as follows: ZMAP4: AUC = 0.944 (sensitivity 95.8%, specificity 55.56%, and cutoff point 1.443); UPP2: AUC = 0.798 (sensitivity 92.6%, specificity 51.85%, and cutoff point 1.345); GALR1: AUC = 0.857 (sensitivity 92.3%, specificity 59.26%, and cutoff point 1.706) (Figure 6B).

## 4 Discussion

In this study, we aimed to investigate the transcriptomic profiles associated with the response to bladder intracavitary HIPEC in high‐risk NMIBC patients. By analyzing gene expression patterns, we sought to gain insights into the molecular mechanisms underlying therapeutic responses. Our findings reveal several key observations that illuminate the response of high‐risk NMIBC to HIPEC. First, we observed distinct transcriptomic alterations following HIPEC treatment, indicating significant changes in the gene expression landscape within the tumor microenvironment (TME). These changes likely reflect the complex interplay between the therapeutic intervention and tumor biology, highlighting the dynamic nature of the response.

Among the notable findings, we identified a set of differentially expressed genes (DEGs) that were consistently upregulated or downregulated in the HIPEC‐treated group compared with the control group. After HIPEC treatment, NMIBC tissue exhibited higher expression levels of TMEFF2, KRT222, IGKV6D−21, GTSF1, IGHGP, AL592429.2, HOXC10, ZIC1, GABRA2, and HMGB1P35. A previous study [13] found that TMEFF2 is a critical factor in modulating one‐carbon metabolism and the invasion of prostate cancer cells. Moreover, TMEFF2 is a crucial biomarker for predicting bladder cancer through DNA‐based analyses of urine samples [14]. These DEGs encompassed various functional categories, including immune response, cellular metabolism, and cell cycle regulation. The dysregulation of immune‐related genes suggests an active modulation of the immune microenvironment, possibly influencing the antitumor immune response and subsequent treatment efficacy.

In the realm of functional enrichment, the Wnt signaling pathway emerges as a pivotal player in conferring resistance to HIPEC in NMIBC. This pathway exerts a crucial influence on various cellular processes, including cell proliferation, differentiation, and migration [15]. Dysregulation of the Wnt signaling pathway has been implicated in several cancer types, including bladder cancer [15]. Activation of the canonical Wnt pathway occurs through the binding of Wnt ligands to Frizzled receptors, leading to the stabilization and nuclear translocation of β‐catenin. Once in the nucleus, β‐catenin interacts with T‐cell factor/lymphoid enhancer factor (TCF/LEF) transcription factors, resulting in the transcriptional activation of target genes involved in cell cycle progression and survival [16].

Several studies [17, 18, 19] have demonstrated the aberrant activation of the Wnt signaling pathway in NMIBC. Elevated expression levels of Wnt ligands, Frizzled receptors, and β‐catenin have been observed in NMIBC tissues compared with normal bladder tissues. This dysregulation is associated with tumor initiation, progression, and resistance to various therapies, including chemotherapy. However, the specific role of the Wnt pathway in NMIBC resistance to HIPEC remains poorly understood.

In addition to the Wnt pathway, our analysis revealed the involvement of specific molecular pathways in the response to HIPEC. Pathway enrichment analysis highlighted the activation of cytokine–cytokine receptor interactions, neuroactive ligand–receptor interactions, and other pathways, suggesting their potential roles in mediating the therapeutic effects of HIPEC [20]. These findings align with the known mechanisms of action of hyperthermia and chemotherapeutic agents, which induce cellular stress, DNA damage, and subsequent cell death.

Specifically, we found higher levels of native B‐cell infiltration and lower levels of activated dendritic cells and monocytes after HIPEC. The relationship between native B cells and chemotherapy in cancer is complex and multifaceted. Chemotherapy can lead to an increase in the number of B cells, which may enhance immune system function [21]. Conversely, B cells can also contribute to the development of chemotherapy resistance. In certain cancer types, B cells can form protective structures known as tertiary lymphoid structures (TLS) within the TME. These TLS can provide a sanctuary for cancer cells, allowing them to evade the effects of chemotherapy and potentially contributing to tumor recurrence or resistance [22]. The decrease in dendritic cells post‐HIPEC is particularly notable, suggesting a potential reduction in antigen uptake and, consequently, a diminished immune response against cancer [23, 24]. Chemotherapy‐induced cell death in cancer cells can release tumor antigens that monocytes subsequently take up and present to other immune cells. However, the observed reduction in monocyte populations after HIPEC indicates that HIPEC might act as a double‐edged sword in shaping the TME of NMIBC.

Finally, we utilized a validation cohort to test the diagnostic efficiency of our findings in NMIBC patients undergoing HIPEC treatment. We found considerable diagnostic efficiency for tissue biomarkers TMEFF2, KRT222, and GTSF1 in predicting relapse in NMIBC patients after HIPEC treatment, as well as ZMAP4, UPP2, and GALR1 in predicting relapse before HIPEC treatment.

Despite the intriguing findings presented in this study, several limitations should be acknowledged. First, the sample size for RNA sequencing and validation was relatively small, and critical baseline information, such as gene mutation characteristics, is lacking. Further validation in larger cohorts is essential to confirm the robustness of our results. Second, the single‐arm design of the study limits our ability to draw definitive conclusions about the causal relationships between transcriptomic alterations and treatment response. Additionally, certain markers require further validation, and their predictive value in assessing treatment response needs evaluation in a larger cohort of NMIBC patients undergoing HIPEC. Therefore, future prospective studies with a randomized controlled design are warranted to substantiate our findings.

In conclusion, our study provides novel insights into the transcriptomic profiles associated with the response to bladder intracavitary HIPEC in high‐risk NMIBC patients. The identified differentially expressed genes (DEGs), molecular pathways, and subtype distributions offer valuable information for understanding the underlying mechanisms of treatment response and may guide the development of personalized therapeutic approaches. Further investigation in larger cohorts and prospective studies is necessary to validate and translate these findings into clinical practice.

## Author Contributions

**Zhicheng Huang:** conceptualization (equal), data curation (equal), formal analysis (equal), writing – original draft (equal). **Tianhui Zhang:** formal analysis (equal), investigation (equal), methodology (equal). **Jinghua Pan:** data curation (equal), formal analysis (equal), funding acquisition (equal), resources (equal). **Guihao Zhang:** conceptualization (equal), data curation (equal). **Linjun Jiang:** formal analysis (equal), investigation (equal), supervision (equal), validation (equal). **Huiming Jiang:** data curation (equal), funding acquisition (equal), investigation (equal). **Pei Wan:** conceptualization (equal), funding acquisition (equal), project administration (equal). **Ying Peng:** conceptualization (equal), data curation (equal), investigation (equal). **Wenchao Zou:** conceptualization (equal), data curation (equal), funding acquisition (equal). **Qinghua Liu:** methodology (equal), project administration (equal), resources (equal), validation (equal). **Nanhui Chen:** conceptualization (equal), data curation (equal), formal analysis (equal).

## Conflicts of Interest

The authors declare no conflicts of interest.

## Supporting information


Figure S1.
Figure S2.
Figure S3.

Table S1.

## Data Availability

Data utilized in the present work can be obtained from the corresponding author on request.
