# Supplementary material for: Transcriptomic Profiles for Elucidating Response of Bladder Intracavitary Hyperthermic Perfusion Chemotherapy in High‐Risk Nonmuscular Invasive Bladder Cancer
Source: Cancer Med. 2025 Feb 20;14(4):e70672. doi: 10.1002/cam4.70672 (PMC11842869; doi:10.1002/cam4.70672)
Supplement: Supplementary file 1 — Figure S1. Figure S2. Figure S3. [file CAM4-14-e70672-s001.docx]

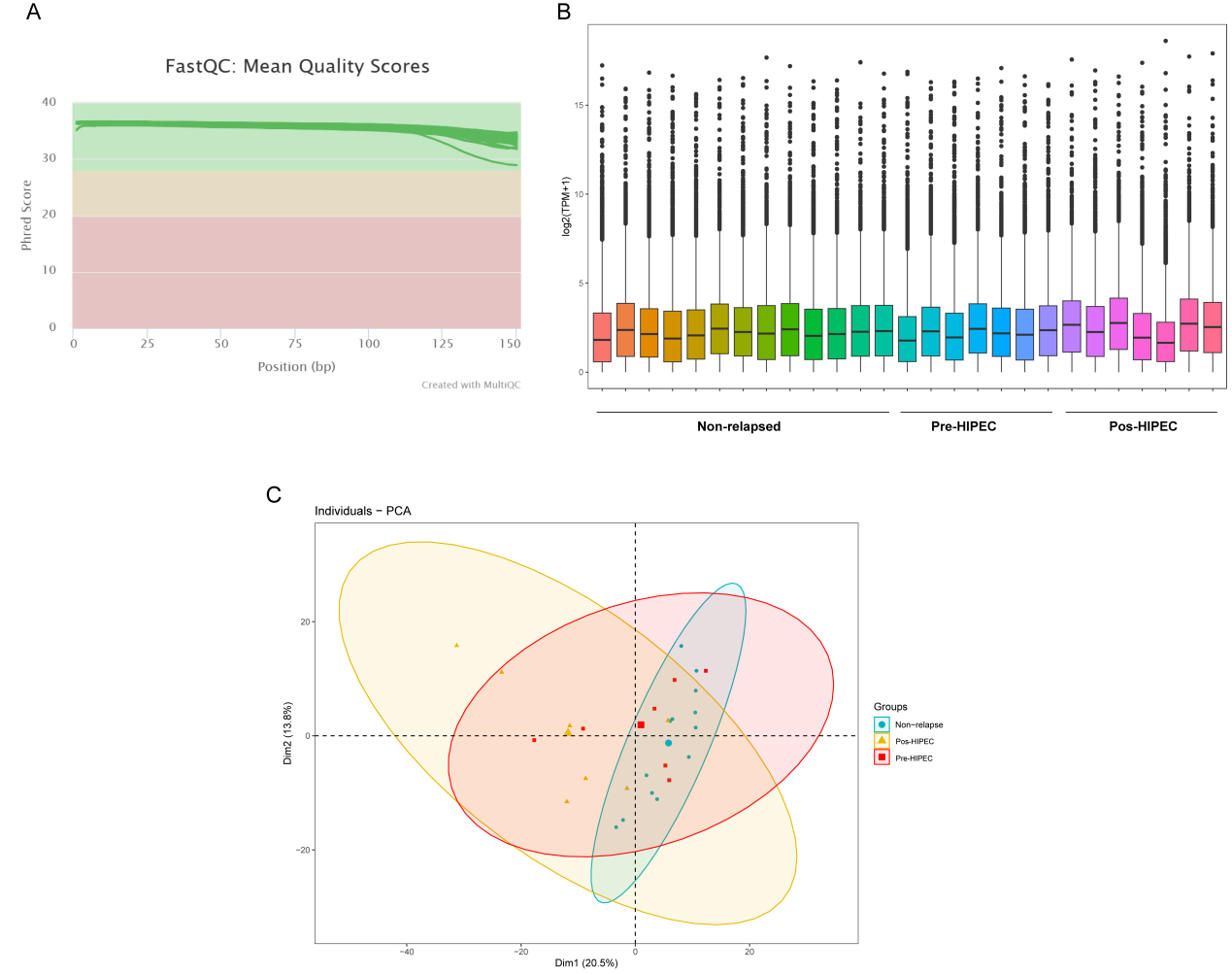


Supplemental Figure 1. the assessment of data quality in the 28 samples. A. the Sequence Quality Histograms of different samples. B. The boxplot for average expression level of 28 samples. C. Principal component analysis (PCA) for the 28 samples.


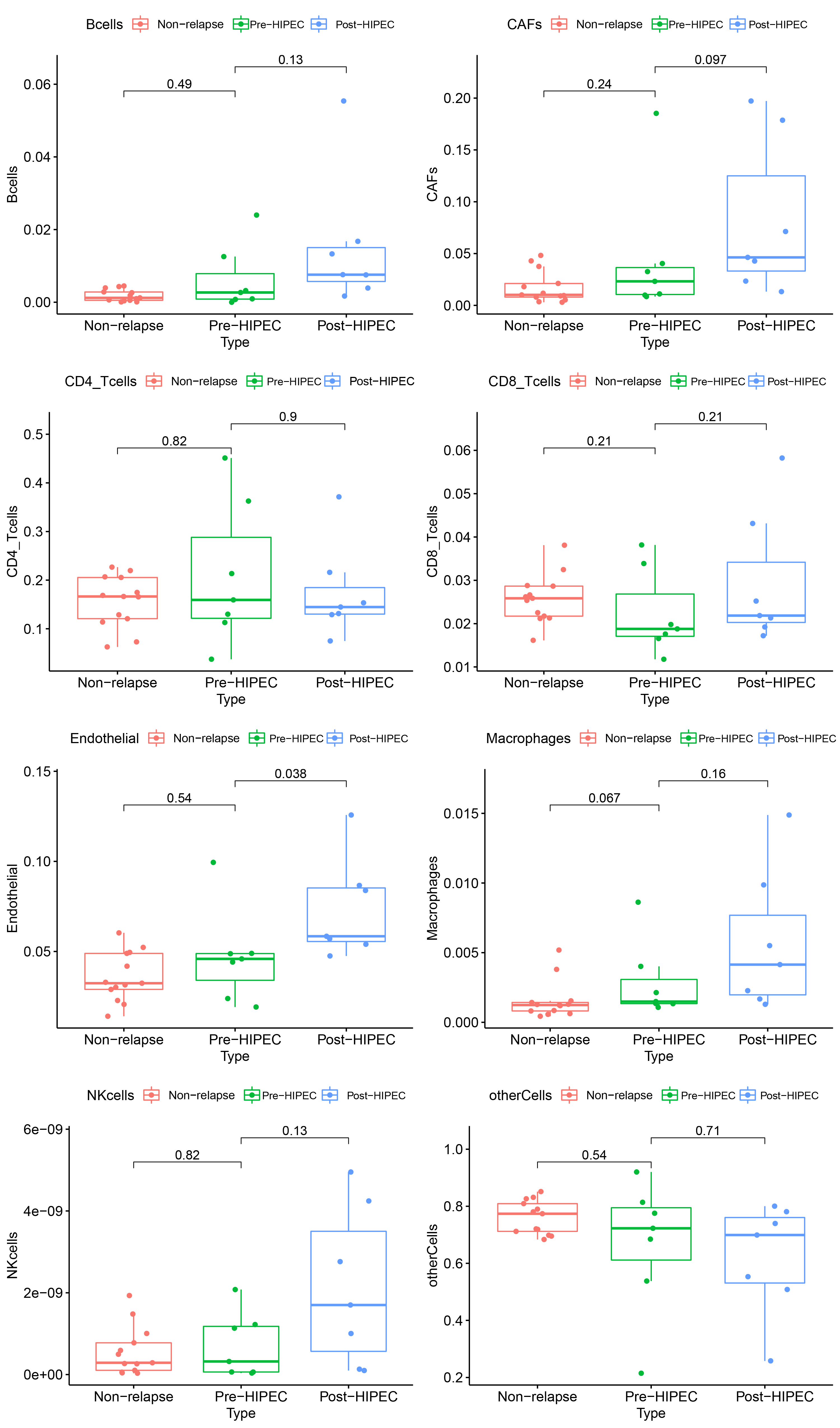


Supplemental Figure 2. Differences in immune cell infiltration scores among different groups via EPIC analysis.


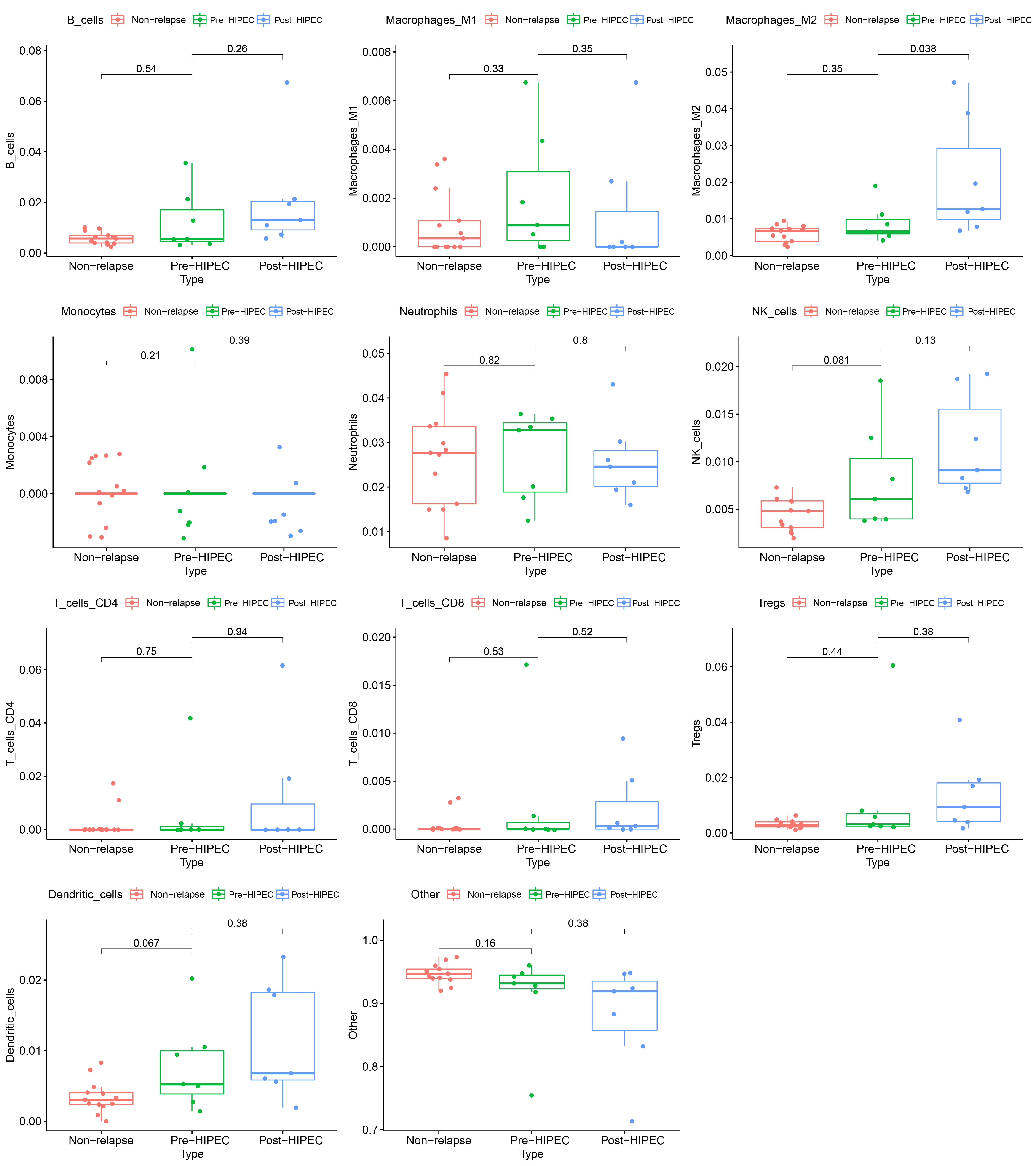


Supplemental Figure 3. Differences in immune cell infiltration scores among different groups via quanTIseq analysis.
