# Supplementary material for: Transcriptomic Profiles for Elucidating Response of Bladder Intracavitary Hyperthermic Perfusion Chemotherapy in High‐Risk Nonmuscular Invasive Bladder Cancer
Source: Cancer Med. 2025 Feb 20;14(4):e70672. doi: 10.1002/cam4.70672 (PMC11842869; doi:10.1002/cam4.70672)
Supplement: Supplementary file 2 — Table S1. [file CAM4-14-e70672-s002.docx]

**Supplementary Table 1: The qRT-PCR primers for this study**

| **Gene** |  | **Primer** |
| --- | --- | --- |
| TMEFF2 | Forward | 5'-CTGCCCGTCATGCTACTCATC-3' |
|  | Reversed | 5'-AATTTACAGGTGTTGGTGTCACA-3' |
| KRT222 | Forward | 5'-GAAATCAGATAGAGACGGTGCTC-3' |
|  | Reversed | 5'-GCTGGCATGTAGGGAGTTTTCA-3' |
| GTSF1 | Forward | 5'-AATCAGGGCTTGCAGGTTTCC-3' |
|  | Reversed | 5'-TGAAGGGACAAGTAGCCAATTTG-3' |
| ZMAT4 | Forward | 5'-ATTCACAGACAGTTACTGCAAGG-3' |
|  | Reversed | 5'-GCATGTTTTCGACTCTCGTAGTG-3' |
| UPP2 | Forward | 5'-GCCTCCAATAGGTCCATGAGA-3' |
|  | Reversed | 5'-ACATTGCTGGTAGGTTGTGTG-3' |
| GALR1 | Forward | 5'-ATCTGCTTCTGCTATGCCAAG-3' |
|  | Reversed | 5'-CAGTGGGCGGTGATTCTGA-3' |
| GAPDH | Forward | 5'-TCGGAGTCAACGGATTTGGT-3' |
|  | Reversed | 5'-TTCCCGTTCTCAGCCTTGAC-3' |
